# 179. Follow-up Blood Cultures for Gram-negative Bacilli Bacteremia Were Associated with Prolonged Length of Hospital Stay and Duration of Antibiotic Treatment: A Propensity Score-matched Cohort Study

**DOI:** 10.1093/ofid/ofab466.381

**Published:** 2021-12-04

**Authors:** Hayato Mitaka, Toshiki Kuno, Shigeki Fujitani

**Affiliations:** 1 Mount Sinai Beth Israel, New York, New York; 2 St. Marianna University, Kawasaki, Kanagawa, Japan

## Abstract

**Background:**

It remains unclear if follow-up blood cultures (FUBCs) for Gram-negative bacilli (GNB) bacteremia should be performed routinely to document clearance of bacteremia due to lack of evidence on the value of FUBCs for GNB bacteremia.

**Methods:**

We conducted a retrospective, multicenter observational study at 4 acute care hospitals to examine if FUBCs are associated with length of hospital stay, duration of antibiotic treatment, and mortality of hospitalized patients with GNB bacteremia. Participants included adult patients who were hospitalized between January 2017 and December 2018 with GNB bacteremia. Patients with and without FUBCs were propensity score-matched with a 1:1. The primary outcomes were in-hospital mortality, length of hospital stay, and duration of antibiotic treatment during the hospital stay.

**Results:**

Of the 442 hospitalized adult patients with GNB bacteremia, 381 were included in the study. Of those, FUBCs were performed in 276 patients (72%). After propensity score matching, we included 87 patients with FUBCs and 87 patients without FUBCs for comparing outcomes. The median length of stay was longer in patients with FUBCs (9 days [interquartile range, 6.0–14.0]) compared with patients without FUBCs (7 days [interquartile range, 4.5–10.5]; P=0.017). The median duration of antibiotic treatment was also longer in patients with FUBCs (8 days [interquartile range 5.5–13.0]) compared with patients without FUBCs (6 days [interquartile range, 4.0–10.0]; P=0.007). No statistically significant difference was observed in in-hospital mortality between patients with and without FUBCs (adjusted odds ratio = 0.37; 95% confidence interval, 0.081–1.36).

**Conclusion:**

Performing FUBCs for GNB bacteremia were associated with prolonged length of hospital stay and duration of antibiotic treatment but not with mortality.

**Disclosures:**

**All Authors**: No reported disclosures

